# Challenges to nursing leadership in research and academia in the UK: A systematic narrative review

**DOI:** 10.1016/j.ijnsa.2025.100411

**Published:** 2025-08-25

**Authors:** Arun Vamadevan, Vijesh Vijayan, KasiReddy Jayasudha, Styja Varghese, Oghale Eboh, Ajeesh Karthikeyan, Christine Cole, Lauren Walker

**Affiliations:** a NIHR Clinical Research Facility, Liverpool University Hospitals NHS Foundation Trust, Liverpool, UK; b University of Salford, Salford, UK; c Stockport NHS Foundation Trust, Stockport, UK; d Sri Sai College of Nursing, Dr. NTR University of Health Sciences, Andhra Pradesh, India; e University of Liverpool and Liverpool University Hospitals NHS Foundation Trust, Liverpool, UK; f Centre for Experimental Therapeutics, University of Liverpool, Liverpool, UK; g Civic Health Innovation Laboratories, University of Liverpool, Liverpool, UK

**Keywords:** Career progression, Clinical academic careers, Nursing leadership, Research capacity, Systemic barriers, United Kingdom

## Abstract

**Background:**

Nurses form the largest professional group in the United Kingdom (UK) healthcare system, yet they remain significantly underrepresented in research and academic leadership. While the medical profession has benefited from well-defined academic pathways, structured support for nurses in research remains inconsistent, limiting their influence in shaping evidence-based care and healthcare innovation.

**Aim:**

This narrative Review explores the current landscape of nursing representation and progression in research and academic leadership in the UK. It identifies enabling factors, systemic and cultural barriers, and the implications for workforce development and policy.

**Methods:**

A systematic narrative Review was conducted using literature published between 2006 and April 2025, sourced from PubMed, Scopus, and the Cochrane Library. Eighteen eligible studies focusing on clinical academic roles for nurses were included. Data extraction and synthesis followed narrative methods, and study quality was appraised using the GRADE framework and ROBINS-I risk of bias tool.

**Results:**

Included studies highlighted a range of initiatives, particularly the HEE/NIHR Integrated Clinical Academic (ICA) Programme, that have supported early clinical academic development and contributed to strengthening professional identity. However, progression to senior research leadership remains constrained by multiple structural and organisational barriers. These include poor visibility of nursing leadership, lack of integrated career frameworks, limited access to research mentors, insufficient protected time for academic activity, and poor alignment between NHS and academic institutions. Cultural expectations, undervaluing of nursing research, and gendered norms around leadership roles further hinder advancement. Key enablers included early exposure to research, access to structured development schemes, inclusive institutional culture, and visible role models. While the overall quality of evidence ranged from low to moderate, the ROBINS-I assessment identified frequent concerns around study design and reporting clarity. Equity issues affecting nurses from global majority backgrounds were underexplored and represent an important area for future research.

**Conclusion:**

Advancing nursing leadership in research requires a nationally coordinated, long-term strategy with sustained investment, equitable access to career development, and cultural transformation. Mentorship, funding, structural support, and inclusive leadership are essential to enabling nurses to fully contribute to healthcare research and innovation. Addressing underrepresentation and inequality is critical for a diverse and sustainable clinical academic workforce.

**Registration:**

Not registered, as this is a systematic narrative Review.

**What is already known on this topic**

• Nurses are the largest group in UK healthcare yet remain structurally underrepresented in research and academic leadership roles.

• Clinical academic pathways for nurses exist in policy but lack national coordination and equitable access.

• Barriers such as limited mentorship, fragmented funding, and unclear progression routes persist despite NIHR-led efforts.

**What this paper adds**

• To the best of our knowledge, this is the first UK-focused review to apply both GRADE and ROBINS-I frameworks in assessing the quality of evidence on nursing research leadership.

• It is also the first to use a systematic narrative Review to critically integrate peer-reviewed and policy literature on clinical academic career progression in nursing.

• This review builds on previous scoping efforts by offering a deeper analysis of the structural, cultural, and equity-based barriers that affect nurses' access to research leadership roles.

• It highlights a national absence of coordinated, equitable academic pathways for nurses, despite longstanding policy commitments.

• To the best of our knowledge, this is the first review to foreground intersectional barriers—such as race, gender, and professional status—within the context of UK nursing leadership.

Alt-text: Unlabelled box

## 1 Introduction

Nurses are central to healthcare delivery, contributing to patient care, advocacy, and clinical innovation. As the largest professional group within the UK healthcare system, they are well-positioned to assume greater roles in research leadership alongside medical and allied health professionals (Storey et al., 2007; Avery et al., 2022). However, their underrepresentation in academic and research leadership reflects more than numerical disparity—it points to a systemic undervaluation of nursing expertise in evidence-based practice, policy development, and scholarly leadership (Sarawad, 2023; American Nurses Association, 2024; Brunt and Morris, 2025). Since the early 2000s, UK policy initiatives have encouraged clinical-academic roles for nurses to bridge practice and research (Ball, Rafferty and Philippou, 2019; NIHR, 2021). Yet, a persistent gap remains between these aspirations and actual progress in advancing nursing leadership (National Academies of Sciences et al., 2021).

In the UK, only 65 registered nurse lead investigators in projects supported by major national funders between April 2017 and September 2022 were traced, and only 55 % responded to a survey on their experiences leading research projects (Farquharson, 2024). Globally, there are nearly 29.8 million nurses, but only a relatively small subset has doctoral-level training and occupy active research roles within academic institutions a pattern mirrored internationally and particularly acute in lower-resourced regions(Yanbing et al., 2021; Bond and Jackson, 2024). This global gap is particularly prominent in low- and middle-income countries, where structural investment in nursing research capacity remains sparse.

Developing robust clinical academic pathways for nurses is increasingly urgent amid escalating healthcare demands and workforce shortages (Buchan, 2009; Shen and Tucker, 2024). Nursing-led research has the potential to address critical clinical challenges, enhance patient outcomes, and contribute to workforce sustainability. As highlighted by Henshall et al. (2021), clinical academic roles strengthen practice-research integration and positively influence care delivery and staff retention (Henshall et al., 2021). Despite the introduction of national initiatives like the NIHR Integrated Clinical Academic (ICA) programmes and regional fellowships, a unified national strategy remains absent. These initiatives often rely on local partnerships, resulting in inconsistent implementation across the UK (Sibbald, Laurant and Reeves, 2006; Burkinshaw et al., 2022). Many nurses remain on the margins of academic leadership due to limited access to resources, inadequate mentorship, and unclear career progression pathways. This raises a critical question: why does nursing continue to lag in academic advancement despite years of policy attention? In contrast, medical research careers in the UK have long benefited from structured pathways such as those recommended by the Walport Report (UKCRC, 2005) fostering physician-researchers with clear routes to leadership. Efforts to build nursing research capacity have been slow, despite longstanding calls for evidence-based practice integration (Smith, 1979; Hopps, 1994; Cowley et al., 2020). While initiatives like Health Education England’s clinical academic programme and Scotland’s strategic policies have driven modest progress, they lack a cohesive national framework. Barriers such as funding constraints, time pressures, and limited senior mentorship continue to obstruct advancement (Marymount University, 2024; Pincha Baduge et al., 2024).

This review examines the status of nurses in UK research and academic leadership, exploring participation trends, enablers, and barriers. Nurse-led research rooted in clinical realities has the potential to inform meaningful change, yet persistent underrepresentation limits its impact and perpetuates generational gaps in leadership. Expanding on Henshall et al.’s (Henshall, 2021) study outcomes, this Review evaluates current evidence to offer strategic direction. Despite being widely respected for their empathy and expertise, nurses remain largely excluded from shaping health research agendas and strategic decision-making(Flaubert et al., 2021). Addressing this imbalance requires coordinated policy reform, institutional support, and a reimagining of nursing’s role in academic leadership.

## 2 Methodology

This review followed the Preferred Reporting Items for Systematic Reviews and Meta-Analyses (PRISMA) 2020 guidelines (Page et al., 2021). A systematic narrative Review approach was adopted to examine nurses’ engagement in clinical academic roles and their progression into research leadership positions in the United Kingdom. This review draws conclusions from diverse evidence sources to evaluate how clinical academic programmes for nurses’ function, identifying key factors that support their success and sustainability. A structured search strategy, rigorous screening, and quality appraisal were applied to Review findings across heterogeneous studies.

### 2.1 Search strategy

Broad and Systematic literature search was conducted across PubMed, Scopus, and the Cochrane Library, covering literature published from January 2006 to April 2025. The search period began with the release of Modernising Nursing Careers (2006), which marked a shift in national nursing workforce policy. Search terms included combinations of keywords such as “clinical academic,” “research nurse,” “nursing research,” “academic leadership,” and “career development,” combined with terms related to healthcare professionals and restricted to the UK. Boolean operators were used to maximise retrieval of relevant literature. Only English-language, peer-reviewed studies were included.

### 2.2 Eligibility criteria

Inclusion criteria:

• Studies that described or evaluated clinical academic roles linking clinical practice and academic settings for nurses.
• Primary research or evaluation reports focusing on nursing career progression in research or academic leadership.
• Studies reporting on interventions, strategies, or outcomes relevant to research capability or leadership development.

Exclusion criteria:

• Studies that focused solely on research activity within clinical settings without academic component.
• Commentaries, editorials, protocols, and abstracts without full-text access.
• Research unrelated to nurses or not conducted in the UK.

Studies that described or evaluated clinical academic roles linking clinical practice and academic settings for nurses were included, even if they varied in methodological rigour. This decision was made to capture policy-relevant and practice-based insights in a field where experimental evidence remains limited. Evaluation reports and grey literature were considered if they provided contextual or implementation-focused data relevant to the review question.

### 2.3 Screening process

Two reviewers (AV and VV) independently screened and evaluated the titles and abstracts identified through the initial search to assess their relevance. Studies that appeared potentially eligible underwent full-text review based on predefined inclusion and exclusion criteria. Any discrepancies between reviewers were addressed through discussion, and a third reviewer (KJR) was consulted when needed to reach consensus. This two-step screening approach was designed to reduce selection bias and maintain consistency in study selection.

### 2.4 Data extraction

Data was extracted into a pre-validated template containing Study design, Sample size, Population, clinical academic role, intervention and reported outcomes with barriers/enablers in Excel by two reviewers (AV and VV). A third reviewer (KJR) verified the extracted data for accuracy, and discrepancies were resolved through consensus within the review team.

### 2.5 Quality appraisal

The researchers evaluated evidence quality from included studies by employing GRADE (Grading of Recommendations Assessment Development and Evaluations) system. GRADE offers a methodical approach to generate transparent ratings regarding evidence certainty that exists between studies about each outcome. Two independent reviewers examined the total risk of bias along with inconsistency and indirectness, publication bias and imprecision for each outcome. The researchers resolved any conflicting assessments by referring to a third reviewer for validation.

### 2.6 Risk of bias assessment

The Cochrane ROBINS-I (Risk of Bias in Non-randomized Studies of Interventions) tool was employed to evaluate the risk of bias in non-randomized studies. Two reviewers independently assessed each included study across relevant domains. For randomized trials, this included evaluation of selection, performance, detection, attrition, and reporting biases. In the case of non-randomized studies, the ROBINS-I tool addressed potential sources of bias such as confounding, participant selection, intervention classification and adherence, missing data, outcome assessment, and selective reporting. Any discrepancies between reviewers were resolved through discussion, with input from a third reviewer when necessary.

### 2.7 Data analysis and synthesis

Researchers determined meta-analysis was not practical due to expected variations in study approaches along with outcome measurement methods. The authors performed a narrative Review due to varying study designs and outcome measures which provided textual interpretation along with context-based analysis of findings. The analysis followed three essential sections according to interventions/strategies which help nurses develop research abilities along with leadership positions while assessing their impact through counts of nurse leaders participating in studies or writing publications as well as subjective assessments of career growth and identifying obstacles and resources which affect nurse research leadership representation. The authors presented data in tables to showcase the study results which revealed patterned data along with inconsistencies and gaps between research.

## 3 Results

### 3.1 Identification of studies

A total of 473 records were identified through database searches across PubMed, Scopus, and the Cochrane Library. After removing duplicates and screening titles and abstracts, 226 full-text articles were assessed for eligibility. Following exclusion based on relevance, language, availability of full-text, and alignment with inclusion criteria, 18 studies were selected for final Review.

**Fig. 1:**
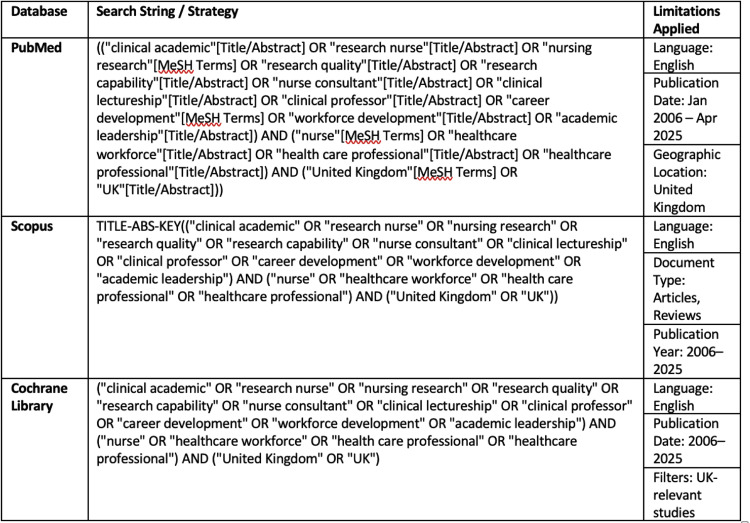
Structured search strategy for identifying UK-based literature on clinical academic career pathways for nurses and NMAHPs (2006–2025).

The study selection process is summarised in the PRISMA flow diagram (Fig. 1).

### 3.2 Characteristics of included studies

**Fig. 2:**
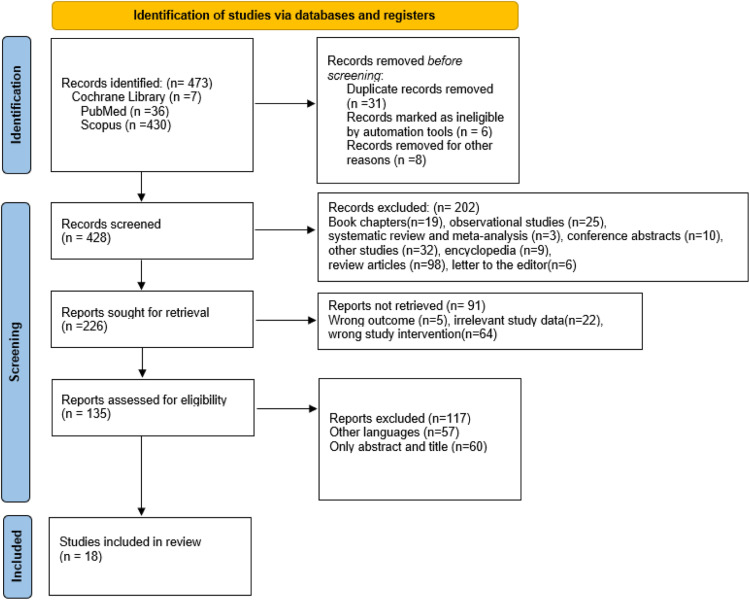
PRISMA 2020 flow diagram detailing the study selection process for systematic narrative review.

The included studies employed a range of research designs, including mixed-method evaluations, qualitative interviews, cross-sectional surveys, case studies, and programme evaluations. Sample sizes varied considerably—from small-scale case studies to national surveys with over 2800 participants—reflecting the diversity of healthcare settings and populations. Most participants were clinical professionals, including nurses and midwives engaged in clinical academic training, particularly through the HEE/NIHR Integrated Clinical Academic (ICA) programme and similar initiatives. The findings consistently demonstrated that formal clinical academic training positively influences participants’ research engagement, professional identity development, career progression, and their ability to bridge the gap between research and clinical practice. The HEE/NIHR Integrated Clinical Academic (ICA) internship and fellowship schemes have played a pivotal role in supporting early-stage clinical academics, fostering research capability, and enhancing career development (Nightingale et al., 2020; Avery, Westwood and Richardson, 2022)Participants reported improvements in research skills, access to development opportunities, and perceived impact on patient care. However, the sustainability and progression of clinical academic roles remain constrained by systemic challenges. A recurring theme across the literature is the absence of a clearly defined research infrastructure. Participants highlighted the scarcity of roles, unstable funding streams, and lack of formal career pathways as major obstacles to long-term progression (Hiley et al., 2019; Roddam et al., 2019; Avery, Westwood and Richardson, 2022). These barriers are compounded by unclear role expectations, limited institutional support, and the poor integration of academic responsibilities within clinical workloads (Gerrish, 2010; Iles-Smith and Ersser, 2019; Cowley et al., 2020) ( Fig. 2).

Work–life balance was identified as a significant concern, particularly for women, who reported difficulties related to caregiving, mobility, and a lack of flexible working arrangements (Trusson and Rowley, 2021). Cultural and institutional barriers—such as gendered assumptions, weak mentorship networks, and a prevailing bias toward medical research—further hinder progress (Dickinson, 2017; Newington, Alexander and Wells, 2022). Research activity within clinical environments is also impeded by a disconnect between healthcare providers and academic institutions, resulting in misaligned priorities and limited stakeholder engagement (Westwood et al., 2018; Marsh et al., 2019).

Although frameworks like Kotter’s Change Model (Bernhardt et al., 2023) and the DINARC toolkit (Iles-Smith and Ersser, 2019) offer structured approaches to change implementation, their success depends on stronger institutional commitment. Overall, clinical academic career development for NMAHPs is shaped by intersecting structural, financial, cultural, and personal challenges. Coordinated investment, cross-sector collaboration, and tailored mentoring are essential to supporting equitable and sustainable career progression. While some included evaluation reports did not specify sample sizes or used non-standardised methods, their inclusion was justified due to the real-world insights they offered on programmatic implementation. Such evidence was clearly marked in the quality appraisal tables, and its limitations were transparently discussed in both the synthesis and the discussion ( Table 1).

**Table 1:** Summary characteristics of included studies evaluating clinical academic career development for nurses and NMAHPs in the UK.

S.No	Study	Study design	Sample Size	Population	Intervention or Programme	Outcomes	Barriers
1.	Nightingale et al., 2020 (Nightingale et al., 2020)	Mixed methods evaluation	104	Nurses, Midwives, allied health professionals	HEE/NIHR Integrated Clinical Academic (ICA) Internship programme	stakeholder perspectives/ progression	• Competitive applications • Undefined career paths • Few mentors • Funding gaps • Work-life conflicts • Weak representation • Time pressures
2.	Newington et al., 2021 (Newington, Alexander and Wells, 2022)	Qualitative semi-structured interview design	20	Healthcare managers and research-active clinicians outside medicine	Not applicable	Combining roles, pushing boundaries, title fit, mindset	• Undefined “clinical academic” roles • Social perception issues • Jargon concerns • Single-centre scope • Differing internal opinions
3.	Avery et al. 2020 (Avery, Westwood and Richardson, 2022)	Cross-sectional online survey	231	Nurses, midwives, allied health professionals	NIHR fellowship schemes	Enablers and barriers to clinical academic career progression	• Research undervaluation • Lack of support systems • Limited job roles • Financial strain • Post-fellowship transition issues
4.	Cowley et al. 2020 (Cowley et al., 2020)	Qualitative semi-structured interviews	17	Clinical academic NMAHPs	Clinical academic master's and doctoral training	Identity transformation and operationalising transformation	• Institutional barriers • Lack of post-training integration • Role assumptions • Absence of long-term impact data
5.	Trusson and Rowley. D. 2021 (D. Trusson and Rowley, 2021)	Qualitative study with online surveys and semi-structured interviews	173	Clinical NHS academic trainees	Clinical academic training	Experiences and challenges of combining training with family life	• Promotion constraints • Pandemic childcare impact • Geographical restrictions • Gender norms • Scheduling conflicts
6.	Roddam. H et al. 2019 (Roddam et al., 2019)	Qualitative evaluation with thematic analysis	162	Aspiring/active clinical academics and managers in nursing, midwifery and AHS	8 workshops to promote clinical academic career pathways	Perceived benefits (building research capacity, capability, and improving patient care), barriers, and enablers	• Job security uncertainty • Regional inequality • Workshop selection bias • Analytical subjectivity
7.	Pattison et al. 2021 (Pattison et al., 2022)	Framework analysis of case studies	7	Clinical nursing professors	Analysis of professorial roles to develop a clinical academic model	Themes of leadership and capacity building with a proposed model	• Limited senior roles • Funding constraints • Poor academic-clinical alignment • Evidence uptake delays
8.	Bernhardt et al. 2023 (Bernhardt et al., 2023)	Descriptive case study	NA	Clinical managers and non-medical research clinicians	Not applicable	Role identity and interprofessional challenges	• Weak research culture • Training access issues • Staff spread across locations • Limited management support
9.	Dickinson et al. 2017 (Dickinson, 2017)	Survey	2840	Scientist, Pharmacist, Allied health care professionals	Fellowships awarded	105 in 2017, compared to 37 in 2009	Not Reported
10.	Gerrish and Chapman (2017 (Gerrish and Chapman, 2017)	Evaluation report	3	Nurses and midwives	Operationalisation of Integrated Clinical Academic framework in NHS Trust	Successful completion of individual programmes over a six-year period, attainment of doctoral and post-doctoral fellowships, and advancement along the clinical academic career trajectory.	• Funding constraints • Poor EBP integration • Scheduling inflexibility • Managerial disconnection
11.	Hiley et al. (2018) (Hiley et al., 2018)	Survey and semi structured interviews	53	Allied Health Professionals, Pharmacists and Healthcare Scientists	West Midlands Clinical Academic Internship Programme (CAIP) and Masters to Doctorate Bridging Programme (MDBP) 2014–2017	Participants who applied to the HEE/NIHR Integrated Clinical Academic programe Participants expressing intent to pursue a clinical academic career upon programme completion	Not reported
12.	Hiley et al., 2019 (Hiley et al., 2019)	3	Case study	Allied Health Professionals, Pharmacists and Healthcare Scientists	Clinical academic career pathways designed for early-career nurses, midwives, allied health professionals, pharmacists, and healthcare scientists (NMAHPPS).	Influence on clinical practice and research responsibilities, along with the number of healthcare professionals who received fellowships.	• Study-time limitations • Patient tokenism • Scarce resources • Networking challenges • Dual-role strain
13.	Iles-Smith and Ersser (2019) (Iles-Smith and Ersser, 2019)	Not reported	Tool evaluation	Post-doctoral clinical academics	NMAHPs	Facilitators and challenges in progressing in academic careers	• Lack of mentorship • Managerial unawareness • Funding shortage • Role marginalisation
14.	Latter et al., 2009 (Latter et al., 2009)	Evaluation report	Not reported	Available to Band 5 and Band 7 nursing roles, as well as clinical chair positions.	Initial clinical academic career programme incorporating doctoral studentships, post-doctoral fellowships, and clinical chair positions.	Implementation of clinical academic pathway	• Role confusion • Cultural resistance • Poor recruitment • Weak institutional buy-in
15.	Marsh et al. (2019) (Marsh et al., 2019)	Evaluation report	Not Reported	Open to all registered nurses	Nurse Clinical Fellowship Programme: nurses undertake BSc or MSc in Clinical Nursing with specialised research routes	Recruitment to Nurse Clinical Fellowship Programme & International nurses who gained UK nurse registration	• Staffing shortages • Difficulty retaining nurses • CPD support gaps
16.	Newton et al. (2017) (Newton, 2017)	Semi-structured interviews and questionnaires	22	Professionals from allied health, pharmacy, and healthcare science backgrounds (NMAHPPS).	Research Fellowship Scheme offered in 2015 and 2016 by Health Education England in collaboration with the Collaborations for Leadership in Applied Health Research and Care (CLAHRC).	Impact of clinical research role	• Communication gaps • Limited fellowship awareness • Trust funding strain • Role adaptation difficulties
17.	(Upton, 2013)	Mixed methods evaluation	46	Nurses, Midwives, Allied Health Professionals, Pharmacists and Healthcare Scientists (NMAHPPS)	Clinical Academic Research Career Programme aimed at expanding applied research, driving enhancements in key service areas, and creating more opportunities for research-focused career development.	NMAHPs achieving research higher degrees over 29 months NMAHPs eligible to take up consultant or senior academic posts over 29 months	• Perception of Isolation • Balancing Work and Study • Financial Sacrifice • Integration of Theory and Practice
18.	Westwood et al. 2018 (Westwood et al., 2018)	Evaluation	Not reported	Clinical academics in nursing and allied roles	Master’s/Doctoral clinical academic training	Career identity changes and real-world implementation	• Misaligned NHS–HEI goals • Lack of research time • Weak leadership support

### 3.3 Quality appraisal

The included studies varied in methodological quality, with overall ratings ranging from very low to high, as assessed using the GRADE (Grading of Recommendations Assessment, Development and Evaluation) framework. While all studies contributed valuable insights into clinical academic pathways for nurses and NMAHPs, many exhibited limitations across multiple domains.

#### 3.3.1 Risk of bias

Most studies demonstrated moderate to serious risk of bias. Common issues included reliance on self-reported data, voluntary participation, and interviewer effects, which increased susceptibility to social desirability bias. Qualitative studies, such as those by Cowley et al. (2020) and Newington et al. (2022), were particularly vulnerable due to small sample sizes and the absence of strategies to mitigate subjectivity. Studies with mixed methods designs, such as Trusson and Rowley (2021), offered stronger triangulation but still carried moderate bias due to recruitment and attrition issues.

#### 3.3.2 Inconsistency

Findings across studies varied in terms of outcomes and reported barriers, reflecting regional differences and the heterogeneity of clinical academic schemes. For example, Roddam et al. (2019) and Pattison et al. (2021) reported divergent institutional experiences of mentorship and infrastructure, limiting the generalisability of their conclusions. However, certain themes—such as limited access to mentorship and unclear role definitions—were consistently reported.

#### 3.3.3 Indirectness

Most studies were directly relevant to the review question, focusing on UK-based nurses and NMAHPs involved in research and academic leadership. However, a few studies had limited external applicability. For instance, Bernhardt et al. (2023) examined a single mental health trust, and Iles-Smith & Ersser (2019) focused specifically on post-doctoral transitions, reducing their general relevance to the broader nursing population.

#### 3.3.4 Imprecision

Imprecision was a significant limitation in several studies. Those with very small sample sizes (e.g. Pattison et al., 2021, *n* = 7; Bernhardt et al., 2023, single case study) lacked the statistical power to support transferable conclusions. In contrast, survey-based studies like Dickinson et al. (2017), which included over 2800 participants, provided more robust and reliable estimates.

#### 3.3.5 Publication bias

The risk of publication bias was generally low, owing to the inclusion of grey literature, internal evaluations, and non-peer-reviewed reports. However, several studies (Roddam et al., 2019) may have been subject to self-selection bias, where participants with strong opinions or positive experiences were more likely to engage.

Many studies fell into the moderate category, particularly those using mixed methods or larger survey samples (e.g. Avery et al., 2022; Upton, 2013). Studies such as Bernhardt et al. (2023) and Latter et al. (2009) were rated low or very low due to limitations across multiple domains. While the evidence base is valuable for mapping current trends, the overall quality points to a need for more methodologically rigorous, large-scale, and longitudinal studies that can inform national policy and support the development of sustainable, equitable clinical academic pathways for nurses in the UK.

A detailed summary of the GRADE quality assessment across included studies is provided in the supplementary file 1.

### 3.4 Risk of bias assessment

**Fig. 3:**
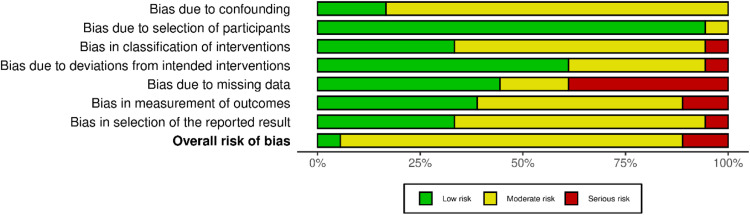
Summary Plot of Risk of Bias Assessments Across Included Studies Using ROBINS-I Domains.

**Fig. 4:**
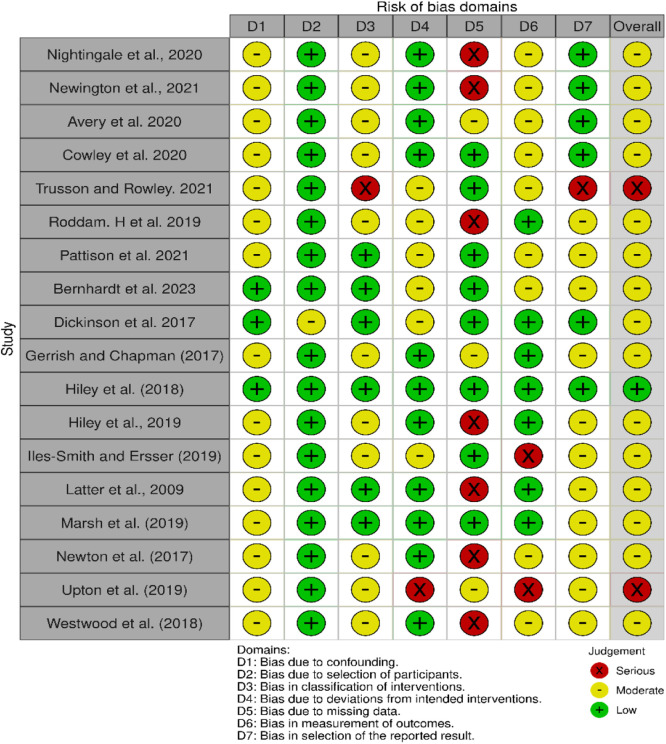
Traffic Light Plot Illustrating Overall Risk of Bias per Study Across ROBINS-I Criteria.

An assessment of risk factors across the included works shows substantial bias within significant elements especially regarding data gaps and protocol implementation deviations. Trusson and Rowley (2021) alongside Upton (2013) display significant risk based on their database evaluation in key domains according to serious bias criteria (Upton, 2013; Trusson and Rowley, 2021). Important weaknesses appear in Hiley et al. (2019) and Newton et al. (2017) due to their serious bias affecting missing data that creates unreliable research outcomes because of participant dropout or incomplete follow-up (Newton, 2017; Hiley et al., 2019). Studies by Bernhardt et al. (2023) together with Hiley et al. (2018) show low bias risk throughout most domains which indicates higher research reliability in their findings (Hiley et al., 2018; Bernhardt et al., 2023). The research participant selection methods demonstrate low risks in studies like Dickinson *et al*. (2017) and Latter et al. (2009) which indicates appropriate selection of study groups thus minimizing external validity concerns (Latter et al., 2009; Dickinson, 2017). A moderate bias exists regarding the classification of treatment interventions and planned clinical procedures throughout the study period according to Cowley *et al*. (2020) and Marsh et al. (2019) (Marsh et al., 2019; Cowley et al., 2020). Such discrepancies in intervention delivery could produce inconsistent study outcomes. Fig. 3, Fig. 4 show the traffic light plot and summary plot for risk of bias assessment.

### 3.5 Career progression and stakeholder perspectives

Stakeholder engagement and institutional support were central themes influencing nurses’ progression in clinical academic careers. The evaluation by Nightingale *et al*. (2020) of the HEE/NIHR Integrated Clinical Academic (ICA) Internship Programme highlighted significant variation in participants’ career trajectories, which often depended on the degree of local organisational support and mentorship available. While some participants reported clear benefits—including increased confidence, research competence, and clinical-research integration—others encountered limited support for advancement beyond the initial stages.

Similarly, Gerrish and Chapman (2017) discussed that nurses who completed ICA programmes successfully progressed to doctoral or postdoctoral fellowships. However, they also documented challenges in transitioning from master’s to doctoral-level study, particularly due to unclear progression pathways and the lack of structured institutional frameworks. The absence of dedicated support systems at key transition points was identified as a barrier to sustained academic development.

Stakeholders, including clinical managers and academic supervisors, expressed a general appreciation for the value of research-active nursing staff. However, this appreciation did not always translate into tangible support. Newington et al. (2022) found that many managers struggled to reconcile academic roles with clinical workforce pressures, resulting in inconsistent prioritisation of research within practice settings. These studies collectively indicate that while formal training programmes provide foundational support, long-term career progression is frequently undermined by institutional inertia, competing service demands, and a lack of coherent leadership strategies to integrate clinical academic roles.

### 3.6 Role identity and career fit

Several studies highlighted the complex and evolving professional identities of nurses and other non-medical clinical academics (NMAHPs) as they navigate dual responsibilities in research and clinical practice. Newington et al. (2022) found that both research-active clinicians and healthcare managers reported significant ambiguity surrounding the definition and expectations of clinical academic roles outside of medicine. Managers acknowledged the value of embedding research within clinical teams but cited workforce shortages, role confusion, and lack of institutional clarity as key barriers to effective integration. Clinicians themselves reported uncertainty in articulating their professional identities, often describing a sense of being “in between” roles. This was especially pronounced in organisations lacking formal recognition or support structures for dual-role professionals. Cowley et al. (2020) found that early-career clinical academics experienced identity transformation through their engagement in research, but this was frequently undermined by institutional cultures that failed to validate or accommodate hybrid roles. Participants described feeling marginalised or misunderstood by both academic and clinical peers.

### 3.7 Training and research pathways

Clinical academic training programmes offer vital early-career support for NMAHPs pursuing research. Trusson and Rowley (2021) found that while trainees valued developmental opportunities, many—particularly women—struggled to balance training with caregiving, mobility limitations, and rigid promotion structures (Trusson and Rowley, 2021). Hiley et al. (2018; 2019) evaluated entry-level and bridging schemes such as the Clinical Academic Internship Programme (Hiley et al., 2018, 2019). These initiatives enhanced participants’ research confidence and fostered interest in academic careers. However, access to further opportunities remained uneven and heavily dependent on institutional culture and leadership support. A persistent challenge was the lack of progression pathways. Participants often faced unclear expectations, limited funding, and insufficient mentorship when transitioning between academic stages. Without sustained organisational backing and protected research time, attrition risk remained high. Early-career researchers together with other professionals indicate facing difficulties while searching for appropriate training programs and research positions according to Bernhardt et al. (2023) (Bernhardt et al., 2023). The Lack of information regarding fellowship programs and research funding results in missed promotion opportunities according to the research by Newton et al. (2017) (Newton, 2017).

### 3.8 Research capacity building and impact

Roddam et al. (2019) and Pattison et al. (2021) examined strategies for developing research capacity among both emerging and established clinical academics (Roddam et al., 2019; Pattison et al., 2022). Roddam et al. reported that participants viewed research engagement as beneficial to both their professional development and patient care (Roddam et al., 2019). However, concerns around job security and disparities in regional research infrastructure were persistent barriers. Pattison et al. highlighted the value of leadership development alongside capacity-building initiatives (Pattison et al., 2022). While such efforts were acknowledged as foundational for embedding research into practice, challenges remained—particularly for senior clinicians—around sustaining research involvement in the later stages of their careers.

### 3.9 Clinical academic career development models

The review identified considerable variation in clinical academic career development models across the UK, with programme design and implementation differing significantly by region and institution. Henshall et al. (2021) reported that the structure and sustainability of these models often depended on local leadership, funding availability, and organisational priorities. Where strong local champions and inter-institutional partnerships existed, models were more likely to support progression and integration of research within practice.

Roddam et al. (2019) noted that clinical academic roles in some areas were underpinned by strategic workforce planning, enabling alignment between research objectives and clinical service delivery. In contrast, other regions lacked formalised frameworks, leading to inconsistent access to mentorship, protected time, and career advancement opportunities. Pattison et al. (2021) and Dickinson et al. (2017) both highlighted the role of embedded academic leadership—such as clinical professors—in sustaining research cultures (Dickinson, 2017; Pattison et al., 2022). These positions were instrumental in mentoring early-career researchers and advocating for structural supports yet remained scarce across most settings. Overall, the findings suggest that while clinical academic career models are evolving, their effectiveness depends on sustained investment, national coordination, and local adaptability.

### 3.10 Structural, institutional and financial barriers

The sustainability of clinical academic careers for NMAHPs continues to be undermined by significant structural, institutional, and financial barriers. Many healthcare organisations lack established procedures for integrating research into clinical practice, making it difficult for professionals to balance academic responsibilities with service delivery (Avery, Westwood and Richardson, 2022). This structural void is particularly pronounced at senior levels, where the absence of dedicated clinical academic posts limits opportunities for long-term progression (Pattison et al., 2022). Healthcare and academic sectors frequently operate with divergent goals and priorities. Several studies reported a lack of strategic alignment between NHS organisations and higher education institutions, resulting in conflicting expectations and limited institutional support for dual roles (Westwood et al., 2018). Clinical academics are often left to navigate these disjointed systems with minimal organisational guidance, contributing to role ambiguity and reduced retention.

Financial constraints further compound these structural challenges. Restricted access to research funding, fellowships, and protected time was a consistent concern across studies (Nightingale et al., 2020). The scarcity of long-term financial investment has led to increased dependence on short-term grants, which in turn creates precarious employment conditions. Roddam et al. (2019) and Gerrish and Chapman (2017) noted that short-term contracts and fragmented funding streams contribute to job insecurity and hinder sustained project development and professional advancement (Gerrish and Chapman, 2017; Roddam et al., 2019).

### 3.11 Work-Life balance and career progression challenges

Engagement in research activities presents substantial challenges for clinical professionals, as it necessitates balancing demanding service obligations. The intensity of clinical workloads often leaves limited time for academic pursuits, thereby impeding both research productivity and career progression (Upton, 2013; Hiley et al., 2018). Many professionals find their research commitments extremely difficult because they must handle personal family and childcare responsibilities. Female clinical academics need to overcome gender expectations and stereotypes as these elements affect their career growth potential and networking capabilities according to Trusson and Rowley (2021) (Trusson and Rowley, 2021). Professionals encounter difficulties during their academic progress from one stage to another especially when transitioning between master’s and doctoral fellowships mainly because of insufficient mentorship (Trusson and Rowley, 2021).

### 3.12 Cultural and organizational issues

Clinical academic roles among non-medical healthcare professionals, such as nurses, midwives, and allied health professionals (NMAHPs), are often characterized by ambiguity and marginalization. Newington et al. (2022) found that these professionals frequently encounter unclear role definitions and limited recognition within both clinical and academic settings, leading to feelings of professional isolation (Newington, Alexander and Wells, 2022). This marginalization is compounded by systemic issues; Gerrish (2010) highlighted that medical research often receives greater cultural and institutional support compared to nursing and allied health fields, creating disparities in access to resources and opportunities (Gerrish, 2010).

## 4 Discussion

Research and clinical academic roles in the UK continue to lack sufficient nurse representation even though, we have spent multiple decades developing clinical academic career paths. Multiple obstacles made up of financial constraints together with structural difficulties as well as cultural elements and individual preferences create barriers for nurses to enter research and academic leadership roles. The HEE/NIHR Integrated Clinical Academic (ICA) Internship and fellowship schemes become successful programs to develop research capabilities together with professional identity and better healthcare results as shown through studies published by Nightingale *et al*. (2020) and Avery et al. (2020) (Nightingale et al., 2020; Avery, Westwood and Richardson, 2022). These efforts show inconsistencies because there is no unified national framework for their execution. Insufficient institutional backing along with local distinctions contribute to program limitations because they make expansion and sustainable outcomes more difficult to achieve. A standard career framework's absence leads nurses to face career uncertainty according to Roddam et al. (2019) because they have few senior mentoring opportunities and examples which results in continuous underrepresentation at upper levels in nursing (Roddam et al., 2019).

Lack of available funding stands as an essential barrier that prevents researchers from obtaining research grants and fellowships and continuous academic appointments. Gerrish and Chapman (2017)(30) together with Pattison et al. (2021) (Pattison et al., 2022) demonstrate how economic conditions push nurses toward accepting short-term contracts which erodes stability and continuous work opportunities in academic settings. Research funding competitions become more challenging because nurses face competition from medical professionals who possess established research frameworks outlined in the Walport Report (2005) (UKCRC, 2005). Westwood et al. *(*2018) show that healthcare institutions remain separated from academic environments according to the review findings (Westwood et al., 2018). Nurses face difficulties in performing research duties along with clinical work due to conflicts between organizational objectives and insufficient integration of clinical and academic tasks and insufficient research time and resources.

Cultural and organizational barriers intensify the existing structural challenges present in this situation. Medical research has traditionally received preferential treatment from healthcare sector authorities according to Gerrish and Chapman (2017), yet this stance relegates nursing expertise and female nurses face double discrimination resulting from gender stereotypes and work-life balance issues because their gender represents many nursing professionals (Trusson and Rowley, 2021). The change resistance tools by Kotter’s 8-Step Change Model and DINARC toolkit (Iles-Smith and Ersser, 2019) show promise to address change resistance, but their success depends on institutional backing which currently does not exist. The review has found that personal obstacles like family demands and restricted mobility affect nurses specifically when they want to advance their training and seek career opportunities elsewhere.

Early-career nurses experience positive outcomes from structured training programs according to the research by Hiley et al. (2018, 2019) which develops their research abilities while building their professional identity (Hiley et al., 2018, 2019). Current research demonstrates that nursing employees continue to face barriers when transitioning into senior positions according to Cowley *et al*. (2020) (Cowley et al., 2020). Moreover, the evidence demonstrates nurses have beneficial clinical perspectives for leading research, yet their research potential stays unutilized because of inadequate policy execution and resource distribution. While training schemes effectively build early capacity, long-term strategies are essential to ensure continuity, integration, and equitable access.

Insufficient mentorship programs together with minimal exposure to nursing research in clinical areas reduces the potential for leadership development because of cultural workplace obstacles. This scarcity of mentorship is especially pronounced for women and individuals from ethnic minority backgrounds, who may face additional barriers due to intersectional factors such as gender and race. Addressing these issues requires a multifaceted approach. Healthcare institutions and academic organizations must work collaboratively to clarify the roles and responsibilities of clinical academics, ensuring that NMAHPs receive equitable support and recognition. Developing structured mentorship programs that are sensitive to the diverse needs of these professionals is essential. Such programs should aim to provide guidance on career development, research opportunities, and work-life balance, thereby fostering an inclusive environment that supports the growth and retention of NMAHPs in clinical academic roles.

The research evidence quality of studied works varies because methodological limitations including small sample groups and self-selection tendencies as well as regional dataset boundaries diminish their broader application. The findings from Dickinson *et al*. (2017) demonstrate clear scientific strength yet ongoing research needs better quality evidence according to studies such as Bernhardt *et al*. (2023) (Dickinson, 2017; Bernhardt et al., 2023). The narrative Review method shows identical patterns of possibilities combined with challenges, yet it does not explain long-term outcomes or intervention effectiveness. Researchers must conduct long-term studies that follow nurses' professional development paths while evaluating how well their clinical academic positions sustain over time and comparing them to how doctors advance in their careers to discover applicable approaches for change. The review demands policy makers to create a fundamental transformation in nursing’s strategic and intellectual role in healthcare beyond modest changes.

While this review focuses on the UK context, the findings have wider implications for international efforts to strengthen nursing leadership in research and academia. Many high-income and middle-income countries face similar challenges: fragmented academic pathways for nurses, structural undervaluation of nursing-led research, and gendered barriers to leadership. The barriers and enablers identified such as the need for integrated mentorship, protected research time, and national coordination, are likely relevant to healthcare systems globally. Moreover, countries aiming to expand research capacity and workforce sustainability can draw on UK-based experiences, both positive and cautionary, to inform the development of inclusive and resilient clinical academic frameworks for nursing professionals.

Global Majority Nurses in the UK encounter multiple challenges that include discrimination and unequal opportunities to advance their careers and scarcity in leadership roles and research participation (Iheduru‐Anderson, 2020a). Systemic racism combines with unfounded preferences and the absence of mentorship opportunities to establish significant obstacles that restrain minority nursing professionals from achieving their best possible healthcare outcomes (Banaji, Fiske and Massey, 2021; Togioka and Young, 2025). Nurses from global majority in the UK face distinct and persistent challenges within both clinical practice and academic progression (Johnson et al., 2021; Grewal et al., 2024). The shortage of visible multicultural leaders and suitable cultural support structures increases the sense of social isolation and undervaluation among healthcare professionals (Iheduru‐Anderson, 2020b; Iheduru-Anderson, Agomoh and Inungu, 2021). The Florence Nightingale Foundation’s Leadership Scholarships and NHS England’s Stepping Up Programme together with the Florence Nightingale Foundation’s Leadership Scholarships represent two specific leadership development programs that focus on empowering global majority staff through mentorship combined with visible opportunities for growth (Rose et al., 2017; Hammond et al., 2022). These findings may also inform similar challenges faced by healthcare systems globally, particularly in advancing equitable nursing research leadership.

A limitation of this review lies in the inclusion of evaluation reports and policy-focused studies that did not report sample sizes or followed non-standard methodologies. While such sources offered important insights into implementation and system-level challenges, they were interpreted with caution. Their inclusion was justified given the limited availability of high-quality empirical studies in this area and is consistent with narrative review methodology.

## 5 Conclusion

This systematic narrative Review indicates that UK clinical academic routes have shown limited success in building research capability and nurse expertise advancement. Nursing researchers encounter persistent structural financial cultural and personal barriers that prevent their academic advancement and leadership roles in academic institutions. The lack of a cohesive national research strategy combined with ongoing underfunding and poor mentoring and institutional backing throughout the early 2000s has restricted nurses from changing their research aspirations into concrete outcomes. The lack of nursing perspectives in research and development for essential health and care research alongside evidence-based practice determination results in diminished healthcare innovation and workforce sustainability for both the nursing profession and patient care advancement. The future of healthcare research requires immediate investment in specific career development structures and sustained financial backing and cultural transformation for nurses to achieve equal involvement.

## CRediT authorship contribution statement

**Arun Vamadevan:** Writing – review & editing, Writing – original draft, Visualization, Validation, Project administration, Methodology, Formal analysis, Data curation, Conceptualization. **Vijesh Vijayan:** Writing – review & editing, Methodology, Formal analysis, Data curation. **KasiReddy Jayasudha:** Writing – review & editing, Data curation. **Styja Varghese:** Writing – review & editing. **Oghale Eboh:** Writing – review & editing. **Ajeesh Karthikeyan:** Writing – review & editing. **Christine Cole:** Writing – review & editing, Supervision. **Lauren Walker:** Writing – review & editing, Supervision, Methodology.

## Declaration of competing interest

The authors declare that they have no known competing financial interests or personal relationships that could have appeared to influence the work reported in this paper.

## Appendix Supplementary materials

Image, application 1
