# Supplementary material for: Challenges to nursing leadership in research and academia in the UK: A systematic narrative review
Source: Int J Nurs Stud Adv. 2025 Aug 25;9:100411. doi: 10.1016/j.ijnsa.2025.100411 (PMC12452846; doi:10.1016/j.ijnsa.2025.100411)
Supplement: Supplementary file 1 [file mmc1.docx]

Supplementary file 1

Table 1: Quality Appraisal of Included Studies Using the GRADE Framework Across Five Bias Domains

| **Study** | **Study Design** | **Risk of Bias** | **Inconsistency** | **Indirectness** | **Imprecision** | **Publication Bias** | **Overall Quality** |
| --- | --- | --- | --- | --- | --- | --- | --- |
| Nightingale et al., 2020 | Mixed methods evaluation | Moderate | Moderate | Low | High | Low | Low to Moderate |
| Newington et al., 2021 | Qualitative interviews | Moderate | High | Low | High | Low | Low |
| Avery et al. 2020 | Cross-sectional survey | Moderate | Moderate | Low | Moderate | Low | Moderate |
| Cowley et al. 2020 | Qualitative interviews | Moderate | High | Low | High | Low | Low |
| Trusson and Rowley. 2021 | Mixed methods | Moderate | Moderate | Low | Moderate | Low | Moderate |
| Roddam. H et al. 2019 | Thematic analysis | Moderate | High | Low | Moderate | Low | Low to Moderate |
| Pattison et al. 2021 | Framework analysis | Moderate | High | Low | High | Low | Low |
| Bernhardt et al. 2023 | Case study | High | High | High | High | Low | Very Low |
| Dickinson et al.  (2017) | Survey | Low | Low | Low | Low | Low | High |
| Gerrish and Chapman (2017) | Evaluation report | Moderate | Moderate | Low | Moderate | Low | Moderate |
| Hiley et al. (2018) | Survey & Interviews | Moderate | High | Low | Moderate | Low | Moderate |
| Hiley et al., 2019 | Case study | Moderate | High | Low | High | Low | Low |
| Iles-Smith and Ersser (2019) | Tool evaluation | Moderate | High | Moderate | High | Low | Low |
| Latter et al., 2009 | Evaluation report | Moderate | High | Low | High | Low | Low |
| Marsh et al. (2019) | Evaluation report | Moderate | High | Low | High | Low | Low |
| Newton et al. (2017) | Interviews & questionnaires | Moderate | High | Low | Moderate | Low | Moderate |
| Upton et al. (2019) | Mixed methods evaluation | Moderate | High | Low | Moderate | Low | Moderate |
| Westwood et al. (2018) | Evaluation | Moderate | High | Low | Moderate | Low | Moderate |
